# The Quebec Semantic Memory Battery: Development, Standardization, and Psychometric Assessment of a Semantic Memory Battery in French

**DOI:** 10.1093/arclin/acae029

**Published:** 2024-04-13

**Authors:** Laura Monetta, Angela Boland, Joël Macoir, Christine L Sheppard, Vanessa Taler

**Affiliations:** École des Sciences de la réadaptation, Faculté de médecine, Université Laval, Québec, QC, Canada; Centre interdisciplinaire de recherche en réadaptation, Université Laval, Québec, QC, Canada; School of Psychology, University of Ottawa, Ottawa, ON, Canada; École des Sciences de la réadaptation, Faculté de médecine, Université Laval, Québec, QC, Canada; Centre de recherche CERVO, Brain Research Center, Université Laval, Québec, QC, Canada; Bruyère Research Institute, Ottawa, ON, Canada; School of Psychology, University of Ottawa, Ottawa, ON, Canada; Bruyère Research Institute, Ottawa, ON, Canada

**Keywords:** Semantic memory, French, Québec, Aphasia, Normative data, Reliability, Validity

## Abstract

**Objective:**

People with aphasia often experience semantic memory (SM) impairment. To improve diagnostic outcomes, SM tasks should recruit various sensory input channels (oral, written, and pictographic), permitting accessible, complete evaluation. There is a need for SM batteries for French-speaking Quebecers that use multiple input channels. The present study, therefore, describes the development of a novel French-language semantic battery: la Batterie québécoise de la mémoire sémantique (BQMS), the assessment of the BQMS’s psychometric properties, and the establishment of normative data for the BQMS.

**Method:**

We first developed eight SM tasks. Following a pilot validation study, we determined the BQMS’s reliability and validity, to ensure consistent, accurate detection of SM impairment. Among French-speaking Quebecers with cerebrovascular aphasia (*n* = 10), people with the semantic variant of Primary Progressive Aphasia (*n* = 4), and healthy controls (*n* = 14), we examined its convergent validity, concurrent validity, test–retest reliability, and internal consistency. Finally, we established normative data for the BQMS by calculating cut-off scores per task that indicate SM impairment (in 93 cognitively healthy French-speaking Quebecers), stratified by sociodemographic variables associated with performance.

**Results:**

The BQMS shows high concurrent, discriminant, and convergent validity, as well as good test–retest reliability and internal consistency. The cut-off score indicating SM impairment ranged from the 2nd to 25th percentiles (stratified by task, age, and sex).

**Conclusions:**

The BQMS’s psychometric properties indicate that it could be a valuable clinical tool for detecting SM impairment. Our normative data will help clinicians detect such impairments.

## INTRODUCTION

Semantic memory (SM) is a subtype of long-term declarative memory (Tulving, 1972) that encodes culturally shared knowledge about objects, facts, places, and people. This includes knowledge of words and their meanings. The brain’s semantic network encodes, stores, and reactivates this information across situations to form concepts (i.e., information we use to make meaning of words, objects, and nonverbal stimuli; Patterson & Lambon Ralph, 2016). According to cognitive models of language, the activation of semantic representations (i.e., concepts) is a central step in spoken and written word comprehension and production (Caramazza, 1997). This step is impaired for people with SM deficits, leading to problems understanding and producing spoken and written words (Suárez-González et al., 2021).

Conceptual knowledge (i.e., SM) is gained through information sources that are specific to various sensory modalities (i.e., sight, touch, hearing, smell, and taste) as well as a “hub” (the anterior temporal lobes; ATLs), representing a critical node in the neural network of semantic knowledge; this hub acts as a convergence area, where semantic information is retrieved, regardless of the sensory input channel (Gorno-Tempini et al., 2004; Lambon Ralph, 2014). In cerebrovascular aphasia (CVA) and in the semantic variant of primary progressive aphasia (svPPA), there is damage to neural networks involved in sensory sources of semantic knowledge; there is also damage to networks devoted to longer-term memory for core representations of concepts (Lambon Ralph, 2014; Patterson & Lambon Ralph, 2016). These semantic impairments in diseases such as svPPA and Alzheimer’s disease generate multimodal deficits (Chapman et al., 2020; Patterson & Lambon Ralph, 2016).

Brain areas damaged in CVA specifically include the left prefrontal cortex and the left temporoparietal region. This type of damage can lead to difficulties accessing semantic knowledge from various sensory channels; it can also produce difficulties using semantic knowledge due to damage to the semantic system (i.e., posterior multimodal and heteromodal association cortex, heteromodal prefrontal cortex, and medial limbic regions; Corbett et al., 2009; Jefferies & Lambon Ralph, 2006; Lambon Ralph & Patterson, 2008; Tippett & Keser, 2022). svPPA involves atrophy of the anterior and inferior temporal lobes bilaterally, with the left ATL being more strongly affected than the right (Gorno-Tempini et al., 2004). Damage to these areas leads to a generalized deterioration of SM, which degrades core semantic representations (within the semantic hub). This damage reduces one’s ability to comprehend and store the meanings of concepts and produce linguistic output (Jefferies & Lambon Ralph, 2006).

Neuropsychological tasks of SM are critical for detecting SM impairment in these acquired cognitive and language disorders; this can improve diagnostic and treatment outcomes. For example, SM symptoms can signal the onset of disorders such as PPA (Gorno-Tempini et al., 2011). It is crucial to develop SM tasks that assess SM using different sensory modalities (i.e., pictographic, spoken, and written) and consider participants’ cultural and linguistic context.

First, tasks that recruit various input and output channels provide a more thorough measure of SM. Because SM is widely distributed throughout the brain, SM deficits can be present in multiple sensory modalities (Corbett et al., 2009; Lambon Ralph, 2014; Senaha et al., 2007). Multiple input and output channels also help ensure that performance deficits result from an SM deficit rather than an oral, visual, or written input deficit (Patterson & Lambon Ralph, 2016). Moreover, tasks that measure SM through various input channels can be administered to people with visual and auditory agnosia.

The cultural context within which tasks are adapted and standardized is a second crucial factor to consider when evaluating SM. Semantic tasks are often language-based and draw from people’s cultural knowledge (Patterson & Lambon Ralph, 2016; St-Hilaire et al., 2016). Therefore, linguistic and cultural differences between populations can produce different SM task results. For example, many items in SM tasks are culture-specific (Callahan et al., 2010; e.g., an association between a windmill and a tulip in the Pyramids and Palm Trees Task [PPTT] is typical to Holland). Items that are not culturally familiar to participants run the risk of Type I error (false positive), where a lack of familiarity causes a low score and is mistaken for a semantic deficit. Furthermore, SM tasks, such as spoken and written, picture-naming assess semantic function through language. Peoples’ linguistic profiles differ significantly depending on factors, such as geographical location and ethnic group. SM tasks should, therefore, be developed, validated, and standardized among diverse populations.

In Canada, the population is aging, and the number of French speakers is rising (Institut de la statistique du Québec, 2017). The rise in French-speakers is primarily accounted for by Quebec, where French is the dominant language and 85.5% of the population speak French at home on a regular basis or all the time (Boberg, 2012; Government of Canada, 2022). Importantly, about 50% of the Quebec population is French monolingual, while only 4% of Canadians in other provinces are French monolingual (Institut de la statistique du Québec, 2017). This stark contrast between Quebec and the rest of Canada highlights the need for a wider range of SM tests tailored to French-speaking Quebecers’ cultural and psycholinguistic profiles. SM batteries that exist in other parts of Canada (e.g., Ohman et al., 2020) should not be used in French-speaking Quebec due to linguistic differences (Boberg, 2012). The linguistic profiles of French-speaking Quebecers also differ from those of people in other French-speaking parts of the world. Quebecois and French people have distinct cultural and linguistic profiles that develop starting in early childhood (e.g., differences in lexicon and word familiarity; Bernicot et al., 1994). Therefore, SM tests for French-speakers from France would not be suitable for French-speaking Quebecers (St-Hilaire et al., 2016).

Currently, speech-language pathologists and neuropsychologists in French-speaking Quebec commonly assess SM using picture naming tests, such as the Boston Naming Test (BNT; Kaplan et al., 1983) and the picture-naming subset of the MT-86 (Béland & Lecours, 1990; Nespoulous et al., 1992). Clinicians also use semantic matching tests, such as the Pyramids and Palm Trees Test (PPTT; Howard & Patterson, 1992; Callahan et al., 2010). More recently developed tests include the Questionnaire Sémantique de Québec (QueSQ; Monetta et al., 2019), the Test de Dénomination de Québec—60 images and 30 images (TDQ-60 and TDQ-30; Macoir et al., 2017; Macoir et al., 2019), the test de la mémoire sémantique liée aux personnes célèbres (POP-40; Benoit et al., 2018), the Batterie d'Évaluation Cognitive du Langage (BECLA; Macoir et al., 2016), and semantic verbal fluency tests (St-Hilaire et al., 2016), among others.

While these tests effectively assess SM, they have limitations. First, none provides a complete measure of SM. The PPTT measures associative semantic knowledge only (i.e., association of related concepts; Callahan et al., 2010), the QueSQ assesses semantic feature knowledge only (i.e., knowledge of concepts’ perceptual and encyclopedic features), and the BNT, TDQ-60, and TDQ-30 assess picture naming only (i.e., stating the name of an image, orally or in writing; Macoir et al., 2017, 2019). The POP-40 also assesses a sole semantic function: answering semantic questions about well-known celebrities in French-speaking Quebec (Benoit et al., 2018). Further, many of these tasks assess SM through only one input modality. For example, the French BNT, the TDQ-60, and the TDQ-30 assess only one input modality (pictures) and one output modality (oral naming); this restricts the use of these tasks among people with visual agnosia and language deficits (Alegret et al., 2009). Additionally, picture naming tasks require language production, an ability that declines significantly in aphasia; these tasks may, therefore, mistake language production impairment for SM impairment. The French PPTT was also normed using only one input modality (pictures) and one output modality (pointing; Callahan et al., 2010). The BECLA, normed for French-speaking Quebecers, measures a wider variety of semantic functions using various input modalities (Macoir et al., 2016). However, there are ceiling effects on most of its tasks for people of certain age groups and educational levels (Macoir et al., 2016), suggesting that the BECLA may not be sensitive to mild SM deficits or changes in SM over time.

Despite the effectiveness of these tests, a short, complete battery is needed for French-speaking Quebecers with acquired language disorders, like aphasia. Such a tool should assess semantic function across multiple sensory modalities. Multiple modalities would permit its use among people with sensory deficits, allowing clinicians to choose a modality most suitable for the patient. This kind of tool should also assess a wide range of semantic functions (e.g., naming, associative matching, and semantic feature knowledge) to thoroughly capture SM in aphasia. Further, it should manipulate and control various psycholinguistic parameters that influence SM as well as different channels of sensory input. Additionally, this tool should be relatively brief to detect SM impairment in a timely manner.

To address this need for a new tool, we developed a French battery designed to assess SM among people with post-stroke and neurodegenerative aphasia: la Batterie québécoise de la mémoire sémantique (BQMS). The BQMS addresses the limitations of existing tests by: being tailored to the psycholinguistic norms of French-speaking Quebec, using a wide range of tasks to assess various SM abilities (e.g., associating words and naming images), evaluating SM in a relatively small number of task trials, and measuring SM using different input and output channels.

In this article, we first describe the development of the BQMS. We also determine the BQMS’s reliability and validity, to ensure consistent, accurate detection of SM impairment. Among French speakers residing in Québec, we examined its convergent validity, concurrent validity, test–retest reliability, and internal consistency. Finally, we established normative data for the BQMS by calculating cut-off scores per task (among cognitively healthy French-speaking Quebecers) that indicate SM impairment, stratified by sociodemographic variables associated with performance. Thus, three studies are presented in this article. In Study 1, we describe the design and development of the BQMS. In Study 2, we report data on the convergent validity, concurrent validity, test–retest reliability, and internal consistency of the BQMS. Finally, in Study 3, we provide normative data for healthy, community-dwelling, French-speaking people residing in Québec. The three studies were approved by the local Research Ethics Boards of the Institut universitaire en santé mentale de Québec (project #218–220).

## STUDY 1: DEVELOPMENT OF THE BQMS

The purpose of Study 1 was to create the tasks and to choose the stimuli of the BQMS, building on the scientific literature and the steps for assessment tool development.

### Materials and Methods

We first created the tasks and chose the battery’s stimuli. We developed eight semantic tasks (each with fewer than 24 trials and 1 practice trial) with items selected on the basis of psycholinguistic parameters that can influence the activation of semantic features; these parameters were taken from the French Lexicon Project (Ferrand et al., 2010) and the Lexique database (New et al., 2001). The French tasks were developed in parallel to a battery of English tasks (Ohman et al., 2020). Some items were shared between the two versions. For example, the English word “mushroom” and the French word “champignon” were both used in the batteries’ picture-naming tasks. Other items were developed for the French version only, according to culturally specific psycholinguistic variables. For example, the action word “écrire” was included in the French naming task only. We administered all tasks in PowerPoint format, on a notebook computer, aside from Tasks 3 and 4, which were, respectively, administered using a multiple-choice sheet and orally. Participants were awarded one (1) point for every correct answer and no points for incorrect answers.

Tasks 1 and 2 were (oral and written) word and image matching tasks (spoken word to image matching and written word to image matching), aimed at capturing knowledge of semantic concepts. In both tasks, participants were presented with four images. They were then instructed to select the image that best represented the word that was orally spoken to them (Task 1) or presented to them in written form (Task 2). For example, if presented with the word “lapin,” (rabbit) participants would choose between images depicting the following: “lapin,” “patin,” “souris,” et “échelle” (rabbit, skate, mouse, and scale). Half of all stimuli for each task were biological (living things) and the other half was artifactual (human-made objects). The stimuli also had equal concreteness (i.e., reference to a perceptible entity) and frequency across categories to prevent confound from overly abstract stimuli. Furthermore, both semantically and phonologically similar distractors (i.e., incorrect response options) were included. For example, the distractor “patin” is a phonological distractor because it sounds like the target word, “lapin.” Whereas the distractor “souris” is a semantic distractor, being categorically related to “lapin” since they are both rodents. There is also an unrelated stimulus (echelle).

Tasks 3 and 5 were associative matching tasks for capturing associative semantic knowledge. Participants were shown a picture (Task 3) or word (Task 5) in a box in the middle of the screen. They were instructed to point to a picture or word that is most related to it (i.e., sharing a contextual link) out of four options in four corners of the screen. For example, for “vase” (vase), the options are “1: arbre,” “2: herbe,” “3: fleur,” and “4: feuille” (tree, grass, flower, and leaf). The correct answer is “fleur.” Task stimuli varied in semantic category (half biological, half artifactual) and had equal concreteness as well as frequency across categories. Distractors are from the same category as the target. For example, the distractors of herbe, arbre, and feuille are within the same semantic category (nature) as the correct answer, fleur.

Task 4 was a Semantic Feature Questionnaire, capturing semantic feature knowledge. This questionnaire is comprised of a series of yes-or-no questions that experimenters orally asked participants about the semantic features of biological and artifact items. For example, for the question: “Est-ce que les singes sont poilus?” (Are monkeys hairy?), participants can select “oui” or “non” (yes or no), with the correct answer being “oui” (yes). The participants were instructed to respond orally. Task 4 controlled for the stimulus feature type being probed (i.e., encyclopedic vs. perceptual knowledge probed by a given stimulus; semantic features probed were equally distributed across items).

Task 6 was a common feature identification task that taps into semantic feature knowledge. In this task, two words were presented to participants, and then they selected the most specific feature that the two concepts have in common from four items presented on a sheet of paper (in multiple-choice format). If presented with the word pair “Léopard–Dalmatien” (leopard, dalmatian) for example, participants choose between: “1: les deux ont des rayures,” “2: les deux sont des insectes,” “3: les deux ont des taches,” and “4: les deux sont des mammifères” (both have stripes, both are insects, both have spots, and both are mammals). The correct answer is “les deux ont des taches.” Stimulus category, concreteness, frequency, and familiarity were matched across stimuli. For each task trial, one distractor is a true shared feature of the two stimuli but within a superordinate or broad category (e.g., leopards and dalmatians are both mammals, but “mammals” is not a specific enough shared feature). Another kind of distractor is superordinate and incorrect; for example, “both are insects” is both broad and not true. The last distractor type for each trial is subordinate (i.e., specific) but incorrect; for example, “both have stripes” is a specific stimulus feature that is not true for leopards and dalmatians.

Tasks 7 and 8 were oral and written picture-naming tasks aimed at capturing concept-naming ability. Participants were presented with visual images of either (i) objects or (ii) people doing various actions. In Task 7, participants orally named the image, and in Task 8, they printed the name of the image on a sheet of paper. The stimuli were different for the oral and written tasks. There was no limit on the number of words participants were allowed to produce in response to these stimuli; however, most correct responses were one word only. Inflections were also counted as incorrect answers, with the exception of one item that had both strict and lenient response options (“[se] faire la barbe”) was accepted as well as the strict response of “[se] raser (shaving”). In Tasks 7 and 8, both semantic (i.e., biological vs. artifact) and grammatical categories (i.e., noun vs. verb) were controlled, as well as frequency and picture complexity. In both Tasks 7 and 8, there were 12 nouns and 6 verbs. All psycholinguistic variables were the same for Tasks 7 and 8, aside from the following: first, in Task 8, orthographic complexity was controlled, to reduce confound resulting from stimuli exceeding participants’ ability to correctly write the word. Further, in Task 8, all verbs were intransitive to facilitate understanding and clarity of stimuli, seeing as stimuli consisted only of one word (that did not require a direct object indicating the person or thing acted upon). Additionally, spelling mistakes were not counted as incorrect answers in this task.

In Tasks 1, 2, 3, 5, and 6 of the preliminary version of the battery, there were 12 stimuli per task. In task 4, there were 24 stimuli. In Tasks 7 and 8, there were 18 stimuli.

Once the tasks and items were chosen, we conducted a validation pilot study with a preliminary version of the battery to develop the final version of the BQMS. This pilot study assessed: the administration protocol for the tasks, the psycholinguistic variables controlled and manipulated, and the instructions for standardized administration and scoring. Clinical experts then reviewed this version to establish the instrument’s face validity. A total of six professionals conducted these assessments (four speech-language pathologists and two neuropsychologists from Quebec, Canada). The professionals reviewed the preliminary BQMS using a validity questionnaire, which provided a global assessment of the battery and of each individual task. Using multiple-choice items and Likert scales, this 16-item questionnaire assessed the BQMS’s appropriateness, usefulness, ease-of-use, as well as the clarity of tasks’ instructions, scoring, and stimuli. Each item had a section for comments below. All informants agreed with the purpose and usefulness of the battery. After their review, we adjusted all test elements where there was a lack of clarity in the instructions or the procedure. For example, to standardize images for naming tasks, the professionals suggested reducing illustrations’ size and using illustrations of the same drawing style. Other changes included making images clearer (e.g., making images of soap and a kangaroo more defined) and more conventional (e.g., choosing an image of a typical-looking tiger).

**Fig. 1:**
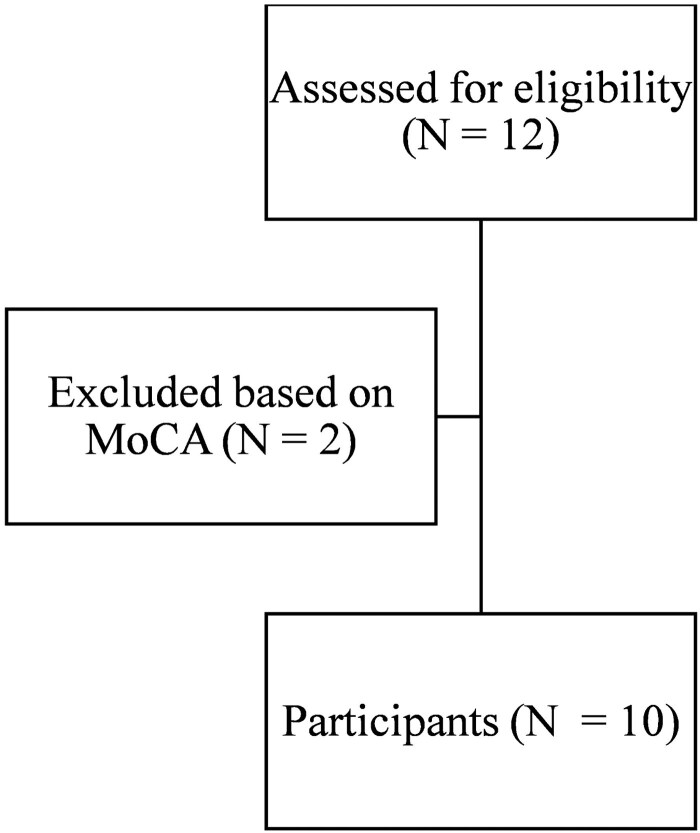
Flow of participants (study 1).

Following the reviewers’ adjustments, we administered the preliminary version of BQMS to a convenience sample of 12 healthy, French monolingual participants, following informed consent. These participants were included in Study 1 only. The purpose of this pilot testing was to examine whether any items may be inappropriate (e.g., overly difficult). The amount of time it takes for healthy participants to complete the battery is 30 min. Participants were recruited from a university [blinded for peer review] and the Quebec community (see Table 1 for participant demographics and neuropsychological characterizations; see Fig. 1 for the flow of participants). Participants did not receive financial compensation.

**Table 1:** Participant demographics and neuropsychological test scores

		**Pilot participants**	**Normative participants**
**Demographic characteristics**	*N*-value	10	**Overall:** 93 (*n* = 27 **Younger adults (YAs):** 19–39; *n* = 21 **Middle-aged adults (MAs**): 40–59; *n* = 45 **Older adults (OAs):** 60–89
	Sex	5 males, 5 females	40 males, 53 females
	Mean age (years)	45.61 (range: 19–74)	55 (range: 20–89)
	Mean education level (years)	14.21 (range: 7–18)	**Overall**: 11.7 (range: 3–17); **YAs:** 12.63 (range: 9–19); **MAs:** 12.33 (range: 7–18); **OAs:** 10.82 (range: 3–20)
**Neuropsychological**			
**test scores**	Mean MoCA score (/30)	28 (range: 26–30)	**Overall:** 27.29 (range: 22–30); **YAs:** 26.96 (range: 22–30); **MAs:** 27.14 (range: 23–30); **OAs:** 27.17 (range: 22–30)
	Mean DTLA score (/100)	94 (range: 81–100)	**Overall**: 93.8 (75–100); **YAs:** 93.81 (range: 83–100); **MAs:** 92.71 (range: 75–100); **OAs:** 94.11 (range: 75–100)
*Mean education difference between normative groups*			
**Kruskal-Wallis *H***	**Df**	***p-*value**	
5.67	2	.058	

*Note:* DTLA scores below 67 indicate linguistic impairment for people with fewer than 11 years of education and 78 for people with 12 or more years of education

### Results

Based on Montreal Cognitive Assessment (MoCA; Nasreddine et al., 2005) scores, participants’ mean score indicated normal cognitive function, aside from two participants who were excluded (final *n* = 10). Based on the Detection Test for Language Impairments in Adults (DTLA; Macoir et al., 2017), participants did not show language impairment either. All participants were tested individually in a quiet room at their home, and the tasks were administered without any time constraints. Participants received the following mean BQMS task scores: Task 1 (Spoken word picture matching): 11.9/12 (*SD* = 0.32); Task 2 (Written word picture matching): 12/12 (*SD* = 0.00); Task 3 (Associative matching: Pictures): 11.9/12 (*SD* = 0.32); Task 4 (Semantic feature questions): 23.7/24 (*SD* = 0.68); Task 5 (Associative matching: Words): 12/12 (*SD* = 0.00); Task 6 (Common feature identification): 11.3/12 (*SD* = 0.67); Task 7 (Spoken picture naming): 12/12 (*SD* = 0.00); and Task 8 (Written picture naming): 7/12 (*SD* = 2.26). Two pictures in the Written Picture Naming Task that participants had difficulty identifying were removed in the final version of the test. See Table 2 for the final BQMS tasks. The BQMS protocol, administration procedure, and instructions are freely available on ResearchGate and upon request from the first author.

**Table 2:** BSQ task information

**Task**	**Input modalities**	**Stimulus properties and trial information**
Tasks 1 and 2: Word-picture matching (oral and written)	Participants complete the task in two modalities: matching spoken words to pictures (Task 1) and matching written words to pictures (Task 2). Participants respond by pointing to the correct response on the computer screen	Stimuli for each task comprise six action items, six artifact items, and six biological items; stimuli do not overlap between Tasks 1 and 2. The stimuli also had equal concreteness (i.e., reference to a perceptible entity) and frequency across category
Tasks 3 and 5: Associative matching (pictures and words)	Task 3 has pictures as the input modality and Task 5, written words. Participants are required to identify the associated image or word by pointing	Each subtask comprises 12 trials, six biological and six artifact items (with no overlap between subtasks)
		
Task 4: Semantic feature questionnaire	Experimenters asked participants spoken questions. Participants verbally respond with “yes” or “no” answers	There were 12 items per category (biological and artifact; 24 total)
Task 6: Common feature identification	Words are presented in written form and simultaneously spoken by the experimenter. The participant verbally responds	The task contains 12 trials: six pairs of biological items (i.e., living things) and six pairs of artifact items (i.e., manmade items)
Tasks 7 and 8 Picture naming (oral and written)	Participants complete the task in two modalities: spoken picture naming (Task 7) and written picture naming (Task 8)	Stimuli for each task comprise six action items, six artifact items, and six biological items; stimuli do not overlap between Tasks 7 and 8

*Note:* In the naming tasks (tasks 7 and 8), we used strict scoring to establish our norms (i.e., only exact answers were accepted as correct). However, based on a common response given by 13% of healthy participants, an alternative response can be accepted as a correct (lenient) answer in the Oral Picture Naming task. Specifically, for the action image “(se) raser,” 12 of the 93 participants in the normative study responded, “faire la barbe.” “Faire la barbe” can, therefore, be considered correct. This response was validated according to accepted lexical variations for concepts taken from a Quebec-French terminology dictionary (“Office québécois de la langue française,” 2012).

## STUDY 2: VALIDITY AND RELIABILITY OF THE BQMS

The purpose of Study 2 was to provide data on the BQMS’ convergent and concurrent validity, its test–retest reliability, and its test–retest and internal consistency. The BQMS was designed to assess semantic impairment in people with aphasia (post-stroke and neurodegenerative). Therefore, convergent validity, discriminant validity, and internal consistency were established by including participants presenting with neurodegenerative aphasia and participants with post-stroke aphasia.

### Materials and Methods

**Fig. 2:**
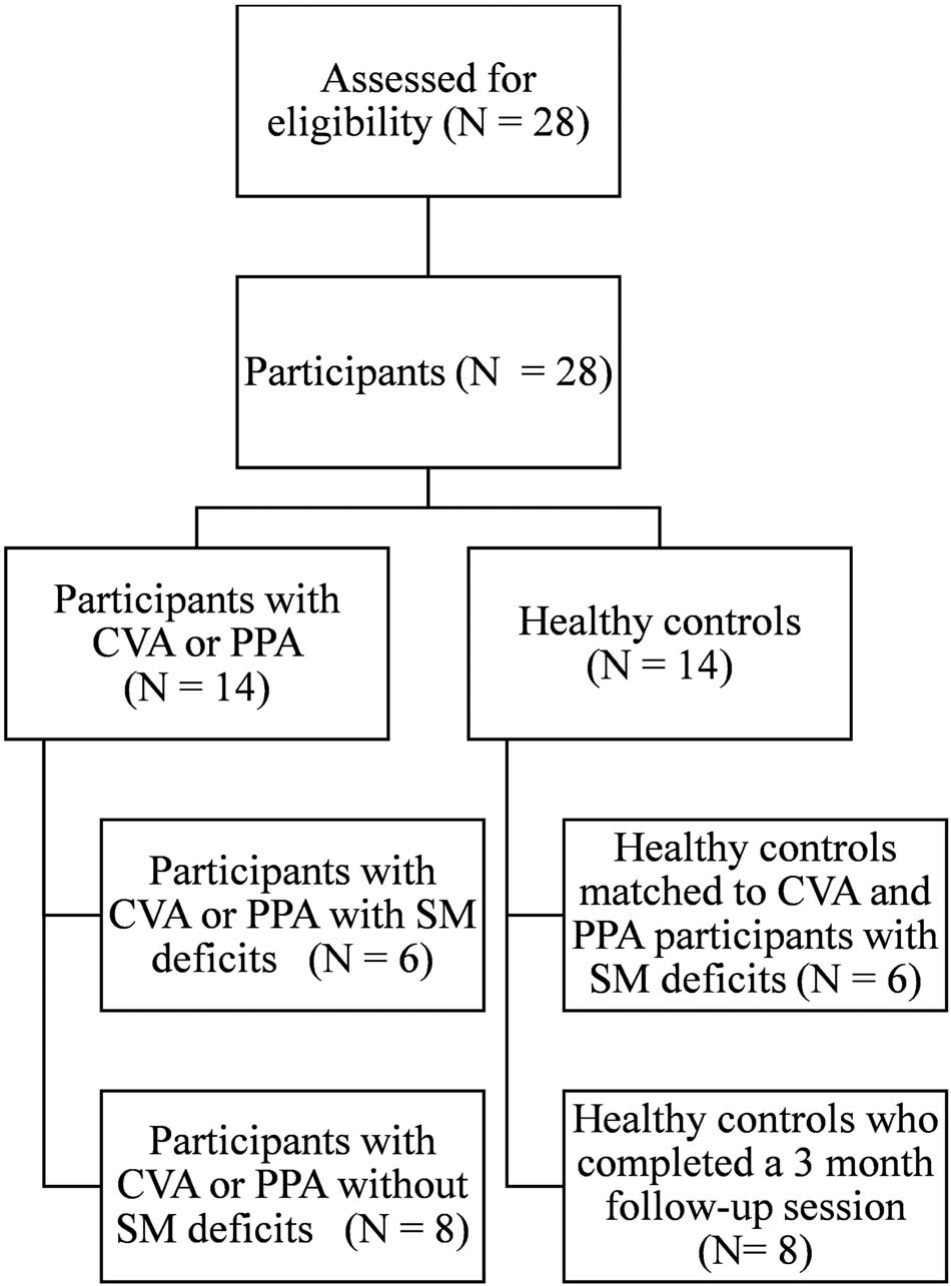
Flow of participants (study 2).

Participants were tested individually in a quiet room at their home or at the research center. We systematically varied the order of task administration across participants. Written protocols for the tests were collected by research assistants, and all tasks were administered without any time constraints. Participants were informed that clinical diagnoses would not be made on the basis of their performance on the present study’s tasks. For our psychometric assessment of the BQMS, we recruited a convenience sample of French-monolingual participants with CVA (*n* = 10) and svPPA (*n* = 4) from an aphasia association (Association des personnes intéressées à l'aphasie et à l'accident vasculaire cérébral ARTERE [APIA-AVC], Québec, Canada) and from our research center database. We also recruited a convenience sample of 14 age-, sex-, and education-matched HCs. The convenience sample of 14 healthy participants was included in both Study 2 and Study 3; participants with aphasia were included in Study 2 only. Based on a health questionnaire administered to participants, six of the participants with aphasia had mild to severe SM deficits (three with CVA and three with PPA). The rest of the participants with aphasia had less severe deficits. We administered the health screening questionnaire, the MoCA, and the DTLA to validate the inclusion/exclusion criteria; scores that are 1.3 standard deviations (SDs) below the normal MoCA range for different ages and genders of middle-aged and older Quebec-French people (Larouche et al., 2016) indicate cognitive impairment. The exclusion criteria for CVA participants included moderate-to-severe speech comprehension disorders that could interfere with understanding instructions. See Table 3 for demographic information. See Fig. 2 for the flow of participants.

**Table 3:** Participant demographics for the psychometric assessment

	Peoplewith lvPPA	Peoplewith CVA	Healthy controls	*p*-value
*N*-value	4	10	14	
Mean age (years)	75.75 (*SD* = 5.74)	62.20 (*SD* = 14.67)	65.64 (*SD* = 13.38)	.935
Mean education (years)	10.00 (*SD* = 2.45)	13.80 (*SD* = 2.10)	12.5 (*SD* = 3.6)	.863
Gender	5 men, 9 women	6 men, 8 women	.699	

*Notes:*  **lvPPA**: logopenic Variant of Aphasia; **CVA**: cerebrovascular aphasia.

Participants completed the BQMS, PPTT, and BECLA in our psychometric assessment, following informed consent. We used Statistical Package for the Social Sciences (SPSS) versus 25 for Mac (SPSS, Chicago, IL) for statistical analyses. The alpha risk was set to 5% (*p* = .05).

### Convergent Validity

This validity was assessed by correlating participants’ BQMS scores with their PPTT and BECLA scores. We chose the PPTT for this analysis because it is the standard test for assessing semantic function among Québecers with aphasia (Callahan et al., 2010). We chose the BECLA because it is a high-quality measure of language and semantic function for French-speaking Quebecers (Macoir et al., 2016). We ran Spearman’s correlations between the picture-matching subset of the PPTT and the BQMS’s picture-based tasks and between the semantic word association subset of the BECLA and the BQMS’s word-based tasks. We chose to run Spearman’s correlations as opposed to Pearson’s due to the nonnormal nature of our data’s distribution. These separate comparisons were made because of pictures and words being different input modalities to the semantic system (Shaul & Rom, 2019); word recognition recruits brain regions devoted to complex lexical processing, while picture recognition involves the activation of visual recognition brain systems.

### Concurrent Validity

For concurrent validity, we measured the BQMS’s sensitivity and specificity as suggested by Loong (2003). Aphasia and control groups were identified as presenting SM deficits or not, based on their PPTT performance. Of the 14 participants with aphasia, the PPTT identified six with SM deficits. To determine the BQMS’s sensitivity (i.e., its correct categorization of people with SM impairment) and specificity (i.e., its correct categorization of people without), we compared the proportion of BQMS task passes and fails between healthy and SM-impaired participants. First, we matched the six participants with aphasia who showed SM deficits to the six HCs who obtained 100% on the DTLA (indicating a lack of SM deficits). Normative BQMS data (discussed in detail in the following substudy) were then used to classify performance as below or within the normal range. Discriminant validity was assessed using the Mann–Whitney U test to distinguish task scores between aphasia and control groups; we used the Mann–Whitney U test as opposed to a *t*-test due to the nonnormal nature of our distribution.

### Test–Retest Reliability

Eight HCs completed the BQMS a second time, 3 months after their initial session. The two measures, Time 1 and Time 2, were compared via a paired Wilcoxon signed-rank test.

### Internal Consistency

We also measured internal consistency (i.e., the degree of interrelatedness among the BQMS subtests) using the Cronbach alpha coefficient.

### Results

With the exception of a few participants whose scores for Tasks 7 and 8 were missing from analyses, we had no missing data.

### Convergent Validity

In terms of the BQMS’s general psychometric properties, there were high positive correlations between performance on the PPTT and the four picture-based BQMS tasks: written word-picture matching (*r* = .70, *p* < .01), picture–picture matching (*r* = .75, *p* < .01), spoken picture naming (*r* = .70, *p* < .01), and written picture naming (*r* = .86, *p* < .01). There were also high positive correlations between BECLA performance and two of the four word-based BQMS tasks: BQMS spoken word-picture matching (*r* = .65, *p* < .05) and semantic feature questions (*r* = .75, *p* < .05). These findings suggest that picture-based BQMS tasks and the PPTT assess the same construct, and two of the four word-based BQMS tasks and the BECLA assess the same construct (SM). This indicates high overall convergent validity for the PPTT and medium overall levels for the BECLA.

### Concurrent Validity

The psychometric assessments revealed the BQMS’s ability to detect SM impairments in aphasia. First, regarding discriminant validity, Mann–Whitney U tests revealed significant differences between the HC and aphasia groups, with medium to large effect sizes for group differences in all tasks except Tasks 1 and 2, indicating high discriminant validity (see Table 4). The percentile cut-offs for our normative data (outlined in Study 3) were then used to determine sensitivity, specificity, and precision. The BQMS correctly identified all six participants who were determined to have SM deficits, according to the PPTT. The sensitivity of the battery is, therefore, 100%, meaning that it is highly capable of detecting SM deficits when they are present. Six out of six people with aphasia failed one or more BQMS tasks, and six out of the six healthy controls passed all BQMS tasks. Six of the six control participants performed above the cut-off for each BQMS task. Furthermore, no healthy controls performed below the cutoff. The specificity of the battery is, therefore, 100%, meaning that it accurately classifies people without SM impairments as such. The BQMS also showed high precision by detecting deficits in six out of six people with SM difficulties (according to PPTT). Fifty percent of participants with aphasia performed below the cutoff for Task 1, 67% for Tasks 2–4, 50% for Task 5, and 100% for Tasks 6–8.

**Table 4:** Discriminant validity for the BSQ tasks

Task	HC mean	Aphasia group mean	HC SD	Aphasia group SD	*p-*value	eta-squared (*n*^2^)
Task 1	11.8	9.1	0.4	1.6	.310	0.162
Task 2	11.6	9.8	0.5	1.4	.065	0.485
Task 3	11.3	5	0.8	3.2	.015*	0.554
Task 4	23	20.5	0.9	2.6	.041*	0.432
Task 5	17	11.6	1.2	2.1	.026*	0.523
Task 6	17.6	12.2	0.5	4.5	.004**	0.688
Task 7	17.3	11.7	0.52	2.2	.002**	0.791
Task 8	17.8	12.2	0.41	4.5	.004**	0.704

### Test–Retest Reliability

Wilcoxon signed ranks tests indicated that HC participants’ performance on all tasks did not significantly differ between T1 and T2 (see Table 5). These results suggest that the BQMS’s test–retest reliability is excellent.

**Table 5:** Test–retest reliability of the BSQ: descriptive statistics

Task (T1 vs T2)	N	Mean T1	Mean T2	*SD*	*p*-value
Task 1	8	12	12	0	1.000
Task 2	8	12	12	0	1.000
Task 3	8	12	12	0	1.000
Task 4	8	23.75	23.75	0	1.000
Task 5	8	12	11.875	0.354	.317
Task 6	8	18	17.875	0.354	.317
Task 7	8	18	17.875	0.354	.317
Task 8	8	17.875	17.875	11.25	1.000
Valid *N* (listwise)	8		8		

### Internal Consistency

For the complete BQMS (120 items across the 8 tasks), we obtained a Cronbach’s alpha coefficient of 0.742 (item means: 14.52; inter-item correlations: 0.276). According to Cortina (1993), a coefficient above 0.7 indicates acceptable internal consistency.

## STUDY 3: NORMATIVE DATA

The purpose of Study 3 was to provide normative data for healthy, community-dwelling, French-speaking people residing in Québec.

### Materials and Methods

**Fig. 3:**
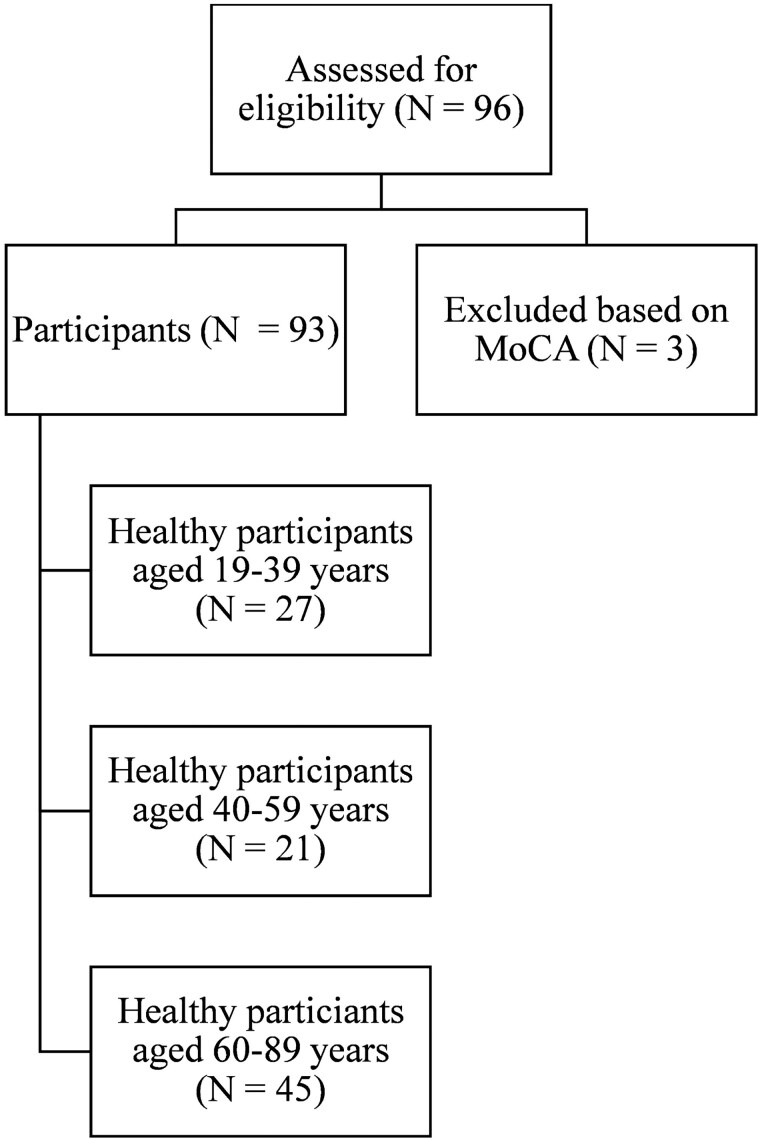
Flow of participants (study 3).

For the normative study of the BQMS, participants were tested individually in a quiet room at their home or at the research center. We systematically varied the order of task administration across participants. Written protocols for the tests were collected by research assistants, and all tasks were administered without any time constraints. Participants were informed that clinical diagnoses would not be made based on their task performance in this study. A total of 96 cognitively healthy French-monolingual adults were recruited using public advertisements for our normative study, where they completed the final version of the BQMS. Before completing the BQMS, participants were screened for eligibility using the MoCA and DTLA. Scores that are 1.3 *SD* below the normal MoCA range for different ages and genders of middle-aged and older Quebec-French people (Larouche et al., 2016) indicate cognitive impairment; three participants scored below this range and were excluded. The final sample thus comprised 93 healthy participants of varying ages, above 19 years. Participants were divided into three age groups (*n* = 27 younger adults: 19–39 years; *n* = 21 middle-aged adults: 40–59 years; *n* = 45 older adults: 60–89 years). See Table 1 for participant demographics and neuropsychological characterization; see Fig. 3 for the flow of participants. We screened participants for inclusion using a health questionnaire, to ensure that they did not have health problems, such as traumatic brain injuries (TBI), psychiatric disorders, or cancer. Participants passed the health screen and had no significant uncorrected visual or auditory deficits or current or past (<3 years ago) speech-language pathology. Participants were not receiving speech-language treatment at the time of testing. As was done in Studies 1 and 2, participants completed informed consent prior to testing.

To reduce the chance of education influencing age-related differences, we first compared younger, middle-aged, and older participant groups’ education levels. Then, using partial Spearman rank correlations, we assessed the relationship between sociodemographic variables self-reported by participants (age and education) and performance scores; participant sex was controlled for in both correlations as well. The influence of sex on task performance was assessed separately using Mann–Whitney U tests. To examine effect size for sex, we used this formula to obtain the eta-squared (*n*^2^) based on the *Z*-statistic: *n*^2^ = *Z*^2^ / *N* – 1. The 1st, 2nd, 5th, 15th, 25th, 50th, and 95th percentiles were calculated for each task (stratified by significant sociodemographic variable[s]), to determine cutoff scores indicating SM impairment. We used SPSS versus 25 for Mac (SPSS, Chicago, IL) for statistical analyses. The alpha risk was set to 5% (*p* = .05).

### Results

In our normative data, all scores had nonnormal distributions (Kolmogorov–Smirnov test: *p* < .05). Additionally, we had no missing data. Due to participants’ wide range of education levels, we first ran a Kruskal–Wallis test to examine age group differences in education. While the *p*-value approached significance (*p* = .058), the educational differences between age groups were nonsignificant. Only the variable of age was correlated with performance on tasks 1 (*r* = −.21, *p* = .05), 3 (*r* = −.37; *p* < .001), 4 (*r* = −.32; *p* = .002), and 7 (*r* = −.49; *p* < .001), with older age being associated with poorer performance. Both age and sex were correlated with Tasks 6 (age: *r* = −.34; *p* < .001; sex: η^2^ = .05; *p* = .04) and 8 (age: *r* = −.30; *p* = .004; sex: η^2^ = .04; *p* = .05); younger adults and females outperformed older adults and males, respectively. There were also sex effects on Task 2 (η^2^ = .04; *p* = .04), where females outperformed males. Education level was not associated with performance on any tasks. Task 5 was not correlated with any of the sociodemographic variables. We chose percentile rank cut-offs for each task (stratified by sociodemographic variables linked to performance). The cut-offs corresponded to 1.5 *SD* below the mean. Thus, a score below the suggested cut-off for a given group is below normal performance. See Table 6 for percentile ranks.

**Table 6:** Percentile ranks stratified by significant sociodemographic variables

		**Mean (Std. error)**	**Percentiles**							
			**1**	**2**	**5**	**10**	**15**	**25**	**50**	**95**
Task1	Age 18–59	12 (0)	12	**12**	12	12	12	12	12	12
	Age 60–89	11.98 (0.02)	11	**11**	12	12	12	12	12	12
Task 2	Females (age 18–89)	12 (0)	12	**12**	12	12	12	12	12	12
	Males (age 18–89)	11.93 (0.04)	11	11	**11**	12	12	12	12	12
Task 3	Age 18–39	11.81 (0.09)	11	11	11	11	**11**	12	12	12
	Age 40–59	11.95 (0.05)	11	11	**11.1**	12	12	12	12	12
	Age 60–89	11.24 (0.14)	9	9	9	**10**	10	11	12	12
Task 4	Age 18–39	23.63 (0.09)	23	23	23	23	23	**23**	24	24
	Age 40–59	23.67 (0.11)	23	**23**	23	23	23	23	24	24
	Age 60–89	22.89 (0.18)	19	19	**20.3**	21	22	22	23	24
Task 5	All age and sex groups	11.73 (0.05)	9	**9.8**	11	11	11	12	12	12
Task 6	Females (age 18–39)	10.87 (0.32)	8	8	**8**	8.6	9.4	10	11	12
	Males (age 18–39)	10.75 (0.25)	10	10	10	10	10	**10**	10.5	12
	Females (age 40–59)	11.07 (0.24)	9	9	**9**	9.4	10.1	11	11	12
	Males (age 40–59)	10.75 (0.25)	10	10	10	10	10	**10**	11	12
	Females (age 60–89)	10.40 (0.23)	8	8	**8.3**	9	9	9.5	10	12
	Males (age 60–89)	9.65 (0.29)	7	7	7	**7.1**	8.2	9	10	11.95
Task 7	Age 18–39	17.81 (0.08)	17	17	17	17	**17**	17.5	18	18
	Age 40–59	17.95 (0.05)	17	17	**17.1**	18	18	18	18	18
	Age 60–89	16.84 (0.19)	14	14	**14.3**	15	16	16	17	18
Task 8	Females (age 18–39)	17.93 (0.07)	17	17	17	**17**	18	18	18	18
	Males (age 18–39)	117.83 (0.11)	17	17	17	17	**17**	18	18	18
	Females (age 40–59)	18 (0)	18	**18**	18	18	18	18	18	18
	Males (age 40–59)	17.75 (0.16)	17	17	17	17	17	**17.25**	18	18
	Females (age 60–89)	17.72 (0.11)	16	16	16.3	17	**17**	17.5	18	18
	Males (age 60–89)	17.5 (0.15)	16	16	16	16.1	17	**17**	18	18

*Note:* Each bolded and underlined percentile rank indicates the cut-off score for a given task that indicates semantic memory impairment. Participants’ education levels were not associated with task scores.

## DISCUSSION

The goals of these three studies were to develop, validate, and norm a complete, yet brief, battery for assessing SM deficits among French-speaking Quebecers with aphasia. Some key advantages that the BQMS has over existing tools are its use of multiple input channels to measure SM and its larger number of semantic tasks. We first created the BQMS’s tasks. Then, we adjusted the battery based on feedback from reviewers to increase the clarity of instructions, procedures, and items. Once the assessments and stimuli were chosen, we conducted a pilot study with a shortened, preliminary version of the battery. We then assessed the convergent and concurrent validity as well as the test–retest reliability and internal consistency of the BQMS. Finally, we established normative data for the BQMS among cognitively healthy, French-speaking adults (younger, middle-aged, and older).

Our psychometric assessment of the BQMS revealed high overall validity and reliability. It, therefore, seems to consistently measure the construct of SM ability in people with aphasia. The psychometric analyses revealed several ways in which the BQMS is equally as useful for capturing SM deficits as other tools, such as the PPTT. This was exemplified by the BQMS’s high convergent validity with the PPTT, indicating that the BQMS meets the standard of the most widely used SM task in French-speaking Québec. Both the BQMS and PPTT also possess high discriminant validity between healthy and SM-impaired groups. We found significant differences between people with aphasia and HCs on all BQMS tasks except for spoken and written word-picture matching (Tasks 1 and 2), likely due to these tasks presenting ceiling effects. The six BQMS tasks that captured declines in people with aphasia likely tap into various semantic degradation processes. For example, the associative matching tasks may capture disruptions in spreading activation (Milberg et al., 1999); the multiple-choice shared feature identification task may capture difficulties accessing specific attributes of concepts (Gravier et al., 2018; Suárez-González et al., 2021); and the naming tasks may capture lexical-semantic and phonological processing deficits (Gravier et al., 2018; Meyer et al., 2016).

In some ways, the BQMS may be superior to existing SM tasks for French-speaking Québecers. First, the battery showed excellent sensitivity in detecting SM deficits. We compared participants with aphasia (identified by the PPTT as showing SM impairments) with HCs on each BQMS task. The six participants who have CVA (with SM deficits) showed poorer performance than HCs on all tasks, indicating high sensitivity. The PPTT and BECLA, on the other hand, have shown ceiling effects among healthy Québecers (Callahan et al., 2010; Macoir et al., 2016). Compared with the PPTT and BECLA, the BQMS may be better suited to detect SM impairments earlier in the course of aphasia and to monitor SM changes over time. The BQMS may also be superior to existing tasks in terms of test–retest reliability. Healthy controls’ BQMS task scores did not differ between the first assessment and after a three-month interval. Scores on the BQMS, therefore, should not vary as a function of testing time. In Klein and Buchanan’s (2009) assessment of the PPTT’s psychometric properties, its test–retest reliability was low, indicating that the PPTT is less useful than the BQMS for measuring SM changes over time.

While the BQMS may be as effective as and, in some ways, more effective than existing tools for assessing SM, it has a few shortcomings. First, despite half of the word-based BQMS tasks being highly correlated with the BECLA, there was no significant correlation between the BQMS and BECLA associative word matching tasks. This finding is counterintuitive due to the tasks’ very similar nature (Callahan et al., 2010) and indicates that the BQMS’s word-based tasks show lower convergent validity than its picture-based ones. However, this may be due to the sample lacking sufficient power to reveal a significant correlation for this task. The BECLA’s ceiling effects on the word association test may also account for this. Another shortcoming of the BQMS is that the word-picture matching tasks (Tasks 1 and 2) did not effectively discriminate between HCs and people with aphasia; this was due to ceiling effects, which were also found in the English versions, where both HCs and people with MCI showed high performance (Ohman et al., 2020). While Ohman et al. (2020) removed these tasks, we plan to include them to detect severe deficits.

We also assessed associations between sociodemographic variables and performance scores on the BQMS. Older age was associated with poorer performance on Tasks 1 (spoken word picture matching), 3 (semantic association of pictures), 4 (semantic feature questions), 6 (common feature identification), 7 (spoken picture naming), and 8 (written picture naming). While these findings are not supported by a wealth of literature showing that SM is resistant to age-related decline (e.g., Martin et al., 2022), other research has revealed an inverse relationship between age and SM (Brickman et al., 2000). For example, age was negatively correlated with associative matching on the French PPTT task (Callahan et al., 2010). Impaired semantic association may arise from age-related weakening of connections between similar concepts (or spreading activation; MacDonald, 2013; Weidler & Abrams, 2018). Both healthy older adults and people with mild cognitive impairment (MCI) have also shown low scores on a common semantic feature identification task (Taler et al., 2020). Younger adults have shown better accuracy and response time than older adults in naming as well, as was observed on the 60-item Chinese naming test (Tsang & Lee, 2003). However, naming declines may also represent degraded word activation within one’s mental lexicon rather than SM deficits.

There were no effects of education for any of the BQMS’s tasks. This finding is consistent with Brambati and colleagues (2012) assessment of the SM system in people with MCI, where there were no associations between educational attainment and semantic performance on famous face recognition tasks. However, the lack of education effects in the present study is inconsistent with the idea of educational bias (as seen in the PPTT), involving older adults with high educational attainment showing increased performance on cognitive tests (Le Dorze & Durocher, 1992); this effect can be driven by cognitive reserve (i.e., resilience to cognitive decline in the face of neuropathology, due to lifetime exposures such as education; Stern, 2012). Educational bias is most often apparent in tests of cognitive domains other than SM (e.g., short-term memory and executive function; Zimmermann et al., 2015). It is, therefore, likely that the BQMS would be a useful clinical tool for older adults with varying levels of education.

There were sex effects in Tasks 2, 6, and 8, suggesting that picture- and word-matching, common feature identification, and written picture naming may be superior in females. These findings are consistent with existing literature showing females to have superior SM (e.g., Loprinzi & Frith, 2018), possibly from more effective semantic processing strategies. Macoir and colleagues (2023) semantic test of video-naming revealed similar patterns, where females outperformed males. However, in most BQMS tasks, there were no sex effects, and those that did show such effects had very small effect sizes. Therefore, males and females showed similar SM performance overall.

We chose the percentiles ranging from the 2nd to 25th as cut-off scores for different BQMS tasks among different sociodemographic groups. Scores below these percentile ranks in each group would be considered below normal performance. Crawford and Garthwaite (2009) suggest that percentile ranks express scores in a way that is more relevant to neuropsychologists than alternative metrics (e.g., mean scores) due to their directly exhibiting the commonality of scores in the normative population.

### Limitations and Future Directions

There are various limitations to consider when interpreting the results. The first limitation is that cultural background was not reported in this study. Considering that SM tasks draw heavily from cultural knowledge (Patterson & Lambon Ralph, 2016; St-Hilaire et al., 2016), it is important that SM tasks be tailored to people from a wide range of cultures. As a next step, larger-scale normative studies and psychometric assessments of the BQMS should be conducted among a larger, culturally diverse sample. Furthermore, participants were presented only with “male” and “female” as sex options in their demographic questionnaire, which could be a source of error. To increase sex and gender inclusivity, open-ended sex and gender questions should be included in the future.

In our psychometric assessment of the BQMS, an additional limitation is that we did not assess divergent validity, which is a key indicator of the battery’s ability to assess the construct of interest (Ivanova & Hallowell, 2013). An important future direction would be to assess the BQMS’s correlations with tests of similar yet distinct constructs (e.g., other language-related functions measured in nonsemantic subsets of the BECLA; Macoir et al., 2019). Furthermore, we only assessed the BQMS’s test–retest reliability among HCs. Consistency in aphasic participants’ performance may vary at longer intervals. For example, people late in the course of the semantic variant of PPA (svPPA) decline by several points per month on the BNT and PPTT, and people with the logopenic variant (lvPPA) decline by one point per month (Sebastian et al., 2018). It may be worthwhile to assess BQMS’s test–retest reliability at different time points among people with aphasia (e.g., at 1, 3, and 6 months), especially neurodegenerative types. Additionally, there were near-ceiling effects for several BQMS tasks. While our clinical sample in Study 2 included some participants with mild SM impairment, larger studies should investigate how people with mild deficits perform relative to those with severe ones.

It is also important to note that performance may have been influenced by generation or educational background. However, even if this were the case, our assessment of people of different age groups and educational backgrounds increases our norms’ representativeness; the normative nature of our data also acts as a guide to interpreting what scores fall within a typical range (despite potential influences from external factors). Finally, despite the task results among our normative sample being highly homogenous, we acknowledge that the size of our samples (for both norms and psychometric assessment) might not have been sufficiently large to ensure representativeness of the French-speaking Quebec population.

## CONCLUSION

This study presented the development, standardization, and psychometric assessment of the BQMS. This eight-task battery was designed considering psycholinguistic parameters that influence performance, namely the nature of semantic traits and input channels for accessing SM. The BQMS will be the first French-normed battery for Quebecers that permits a brief yet complete evaluation of SM and employs different input and output channels.

We established percentile rank cut-offs for all BQMS tasks, many of which differ depending on participant sex and age (or both). The BQMS’s psychometric properties are largely consistent with those of the English battery. In many ways, the BQMS also appears to be equally effective as existing tools for assessing SM. The BQMS showed high convergent validity with the PPTT, meeting the standard of the most widely used test in French-speaking Quebec. The BQMS tasks successfully detected SM deficits, as was shown by its high concurrent and discriminant validity. Furthermore, the BQMS showed high test–retest reliability among HCs after a 3-month interval as well as high internal consistency. However, there was low convergent validity for the BQMS’s two associative matching tasks; higher-powered studies should revisit this to determine the usefulness of these tasks. There were also ceiling effects for the word-picture matching tasks, only to be used when screening for severe SM deficits.

The BQMS’s psychometric properties exhibit that it could be a valuable clinical tool for detecting SM impairments among French-speaking Québecers with aphasia. The norms we established from French-speaking Quebecers will be useful for speech and language pathologists and neuropsychologists to detect such impairments. Using multiple input channels to assess SM, this tool would be accessible to older adults with visual and auditory agnosia, dyslexia, or writing difficulties.

## Funding

This work was supported by the Alzheimer Society of Canada Quality of Life Grant [18-21]. None financial or nonfinancial interest has arisen from the direct applications of our research.

## Conflict of Interest

None declared.
